# Integrating smoking cessation support during lung cancer diagnostic workup: a pragmatic, multicenter, cluster-randomised controlled trial

**DOI:** 10.3389/frhs.2025.1696454

**Published:** 2025-12-09

**Authors:** Ingeborg Farver-Vestergaard, Kaare Bro Wellnitz, Ole Hilberg, Morten Borg, Helle Marie Christensen, Uffe Bodtger, Niels Lyhne, Marie Lavesen, Maria Ralli, Anders Løkke

**Affiliations:** 1 Department of Medicine, Lillebaelt Hospital, Vejle, Denmark; 2 Department of Regional Health Research, University of Southern Denmark, Odense, Denmark; 3 Research Clinic for Functional Disorders and Psychosomatics, Aarhus University Hospital, Aarhus, Denmark; 4 Department of Clinical Medicine, Aarhus University, Aarhus, Denmark; 5 Department of Respiratory Medicine, Odense University Hospital, Odense, Denmark; 6 Department of Clinical Research, University of Southern Denmark, Odense, Denmark; 7 Respiratory Research Unit, Department of Medicine, Zealand University Hospital, Roskilde/Næstved, Denmark; 8 Department of Respiratory Diseases and Allergy, Aarhus University Hospital, Aarhus, Denmark; 9 Department of Respiratory Medicine and Infectious Diseases, Bispebjerg Hospital, Copenhagen, Denmark; 10 Department of Internal Medicine, Hospital Southern Jutland, Sonderborg, Denmark

**Keywords:** addiction, nicotine, respiratory disease, cancer, implementation

## Abstract

**Background:**

Smoking cessation at or around the time of lung cancer diagnosis is associated with improved treatment outcomes, enhanced quality of life and increased survival. However, many patients continue smoking post-diagnosis.

**Aim:**

This study evaluated the effectiveness of a national initiative in Denmark that integrated smoking cessation support into the diagnostic workup for lung cancer within a pragmatic, multicenter, cluster-randomised controlled trial.

**Methods:**

Nine Danish hospitals were cluster-randomised to either the intervention group (integrated cessation support) or the control group (usual care). The intervention was implemented in five hospitals. Eighty-six patients (intervention = 39; control = 47) who were active smokers at referral completed questionnaires assessing smoking cessation initiation, motivation, quality of life and psychosocial consequences of diagnostic workup at baseline and 6-weeks follow-up. Logistic and multiple regression analyses were conducted. Additionally, 140 healthcare professionals completed a survey on cessation support practices pre-intervention, and 54 completed it post-intervention. Descriptive analyses were used to assess changes in clinical practice.

**Results:**

There were no statistically significant differences in smoking cessation initiation between the intervention and control groups (OR = 0.81 [0.41, 1.58], *p* = 0.53; adjusted OR = 0.79 [0.35, 1.79], *p* = 0.57). Among healthcare professionals in the intervention group, a larger proportion reported they “almost always” provided cessation after the implementation (35.1%) than before (18.3%). But the proportion who responded that they “almost never” provide support was also considerably larger after the implementation (13.5%) than before (3.2%). In the control group, proportions tended to shift more generally towards providing more support over time, and a considerably larger proportion reported to refer patients to external smoking cessation support at the follow-up measurement.

**Conclusion:**

The study was inconclusive, showing no significant effect of smoking cessation support during lung cancer diagnostic workup on patients' cessation initiation, possibly influenced by selection bias and varying intervention fidelity at study sites.

## 1 Introduction

Lung cancer is the leading cause of cancer-related death worldwide (1, 2). The disease often progresses rapidly, with a 5-year survival rate of below 25% (3). As the lung cancer progresses, patients frequently experience debilitating symptoms such as breathlessness, pain, fatigue and impaired functioning (4). While surgery, stereotactic body radiotherapy and ablative procedures offer curative potential, most other oncological treatments, including palliative radiotherapy, immuno- and chemotherapy offer life prolongation, symptom management and improved quality of life.

Smoking plays a central role in lung cancer development and subsequent progression. In countries with relatively high smoking prevalence, up to 90% of lung cancer incidents can be attributed to tobacco smoking (1). Smoking cessation at or around the time of diagnosis has been associated with improved treatment outcomes (5), enhanced quality of life (5, 6) and a ∼30% increase in survival rate (7). The period surrounding lung cancer screening and/or diagnosis is often considered a “teachable moment” when patients may be more receptive to smoking cessation advice (8–10). Despite this, smoking cessation remains challenging due to nicotine dependence and psychosocial factors, and studies suggest that up to 50% of patients continue smoking after diagnosis (11, 12).

However, the provision of smoking cessation support in clinical practice is limited. A registry-based study from the United States, reported that only 36% of lung cancer patients received smoking cessation support (13). In Denmark, only 25% of patients who died within a year of diagnosis had stopped smoking, compared to 40% of those who survived beyond the first year (14). Additionally, surveys among healthcare professionals indicate that nearly half rarely or never advice their patients to quit smoking (15). Barriers to providing smoking cessation support at the hospital include time constraints, perceived lack of competence, concerns about damaging the patient-provider relationship, and beliefs that smoking cessation may deprive patients of an effective coping strategy at a difficult point in life, or that it is irrelevant for patients with a poor prognosis (16–18).

In response to these gaps, we launched a national initiative to integrate smoking cessation support during lung cancer diagnostic workup.

## 2 Aim

This pragmatic, multicentre, cluster-randomised controlled trial evaluates the effectiveness of integrating smoking cessation support during lung cancer workup on:

1. Patients’ smoking cessation initiation, motivation to quit, quality of life and psychosocial consequences of workup;
2. Healthcare professionals’ self-reported provision of smoking cessation support, referral to external smoking cessation programmes, as well as their self-perceived competencies, resources and motivation to deliver smoking cessation support.

## 3 Methods

The present study was registered with Clinicaltrials.gov (NCT05192031) and approved by the data authority of the Region of Southern Denmark (21/24984). All participants provided informed consent. The study was exempt from ethical committee approval by the Regional Committees on Health Research Ethics for Southern Denmark (S-20200125) in accordance with Danish law.

### 3.1 Design and setting

The study was conducted in the period from March 2022 to June 2024. In Denmark, nine regional hospitals have the capacity to perform full diagnostic assessment of lung cancer. Several smaller, local hospitals can carry out parts of the assessment, but patients are often referred to regional centres for a complete workup. For this study, we included all nine regional hospitals, as they were selected based on their ability to perform comprehensive lung cancer diagnostics as well as their patient flow in order to ensure an adequate participant inclusion rate. The nine diagnostic centres were randomised to either the intervention group, implementing smoking cessation support as part of the diagnostic procedures and consultations, or the control group, providing care as usual. We aimed to recruit 37 active smokers per hospital to evaluate patient outcomes. Surveys were distributed among healthcare professionals in the participating hospitals to assess provider outcomes.

### 3.2 Procedures and eligibility

Implementation and data collection were piloted for a duration of 2 months at Vejle Hospital. After the first 2 months, the inclusion flow at Vejle was deemed satisfactory, and data collection was subsequently expanded to the other sites without making changes to the recruitment and data collection procedures. Prior to initiating data collection, the remaining eight hospitals were randomly assigned to either the intervention or control group. The intervention was rolled out consecutively at the participating hospitals, with data collection starting alongside implementation. Cluster-level randomisation was chosen to (1) limit training effects to intervention hospitals and (2) minimise contamination bias among patients undergoing diagnostic workup at the same hospital. Following the pilot period at Vejle Hospital, supporting materials to complement communication about smoking cessation with patients were incorporated into the intervention, both for the remainder of the data collection period at Vejle and at the other intervention sites.

Patients were invited to participate and complete the baseline questionnaire through an online link included in their electronic hospital invitation letter before their first assessment visit at the diagnostic centre. Hence, the actual diagnosis of the patients were not yet known at the time of inclusion and at completion of baseline questionnaires, but patients were aware that a lung cancer diagnosis was suspected. Eligible patients were aged >18 years and capable of understanding and responding to electronic questionnaires in Danish. Only patients identified as active smokers (i.e., answering “Yes” to the question “Do you currently smoke?”) at baseline were included in the analyses. Written study information was attached as an appendix to the invitation letter along with a link to the baseline questionnaire. All clinical, diagnostic assessment procedures were completed within a period of 30 days upon referral to the diagnostic centre. Therefore, follow-up questionnaires were distributed at 6 weeks after baseline to ensure that all patients had completed their diagnostic assessment at follow-up.

Healthcare professionals at all participating hospitals were invited to complete an online survey before intervention implementation and again after the implementation period.

### 3.3 Sample size calculation

Prior to study initiation, we estimated that 40% of patients undergoing diagnostic workup would be smokers at the time of inclusion (11, 14). Assuming a 30% increase in the attempt-to-quit rate in the intervention group and accounting for an expected 15% dropout rate, we aimed to recruit a total of 828 respondents, out of which we expected 331 to be active smokers. This resulted in a projected minimum of 37 patients (active smokers) per cluster.

### 3.4 Intervention

The intervention was based on the existing national, evidence-based clinical guidelines for smoking cessation support in lung cancer care (19). Healthcare professionals at intervention sites participated in a group-based educational programme designed to integrate smoking cessation support organically within the existing diagnostic assessment pathway. The programme emphasises tailoring the intensity and timing of support across assessment visits within the diagnostic period (maximum 30 days per patient). Given variations in patients' motivation, communication preferences and diagnostic trajectories, the intervention was intentionally flexible rather than standardised, which allowed adaptation to individual patients' needs. The educational programme was inspired by existing, evidence-based approaches in the field (20) and consisted of (1) training of healthcare professionals and (2) a catalogue of written resources developed specifically for patients undergoing lung cancer diagnostic workup.

#### 3.4.1 Training of healthcare professionals

Healthcare professionals (physicians, nurses, administrative staff) with patient contact attended a 1-hour educational session facilitated by authors IFV and AL. The session covered the latest evidence on the benefits of smoking cessation and the risks of continued smoking in the context of lung cancer and its diagnostic workup. Furthermore, healthcare professionals at the intervention sites were trained in making referrals to external, community-based smoking cessation programs via electronic patient records and encouraged to discuss local barriers and facilitators for providing cessation support. As many healthcare professionals as possible at the intervention sites participated in the training. Management and participants were instructed to share the newly acquired knowledge and procedures with staff who did not attend, and to establish peer-to-peer training processed for onboarding new employees within the clinic.

Nurses at intervention sites participated in an additional 2-hour workshop focused on motivational interviewing principles (21) and the application of the Stages of Change model (22) to assess and support patients' readiness for smoking cessation. The workshop included case-based discussion and peer interaction based on active learning principles (20).

After 1–2 months of clinical practice application, a follow-up workshop (1.5–2 h) was held to share experiences and refine smoking cessation support practices. Nurses were also offered optional peer supervision facilitated by a clinical psychologist to support ongoing learning and integration of smoking cessation support in practice.

#### 3.4.2 Resources to support smoking cessation

A catalogue of written resources was developed specifically for patients undergoing lung cancer diagnostic workup, including:

- An overview of smoking cessation support principles during diagnostic workup (Supplementary Figure S1).
- A patient information leaflet on the benefits of smoking cessation and the risks of continued smoking in the context of lung cancer (Supplementary Table S1)
- A directory of local community-based smoking cessation programs
- A referral note template for electronic patient records (Supplementary Table S2).

Healthcare professionals were encouraged to familiarise themselves with these materials and adapt them for use in their clinical practice during training sessions.

### 3.5 Data collection

Patient-reported outcomes were measured via electronic questionnaires. The primary outcome was smoking cessation initiation at 6 weeks after baseline (post-diagnostic workup), defined as either a positive response to the question “Are you currently in the process of quitting smoking?” or a negative response to “Do you currently smoke?”. Motivation to quit, perceived importance of quitting and perceived capability of quitting were assessed using three 0–100 visual analogue scales (VAS). Quality of life was evaluated using the SF-36 Health Survey (23), providing physical component (PC) and mental component (MC) scores. Psychosocial consequences of the diagnostic workup were measured using the Consequences of Screening—Lung Cancer Questionnaire (24), including subscales for Anxiety, Behaviour, Dejection and Negative impact on Sleep (higher scores indicating higher impact of diagnostic workup) as well as Relaxed/calm, Social relations, and Existential consequences (higher scores indicating higher impact of diagnostic workup).

Baseline questionnaires also collected sociodemographic and smoking-related data, including age, gender, number of cigarettes smoked per day, age at smoking initiation, number of quit attempts, living with a smoking partner (yes vs. no) and nicotine dependence using the Fagerström Test for Nicotine Dependence (25).

Healthcare professional-reported outcomes were collected through an online survey before and after the implementation period. The survey link was distributed among all healthcare professionals employed in the participating hospitals at the time of distribution. The samples before and after the implementation period, respectively, may therefore consist of different individuals (independent samples). The survey gathered data on age, gender, job type (physician, nurse), years of employment and healthcare experience. Frequency of smoking cessation support activities (assessing smoking status, providing cessation advice, offering referral information) was measured on a 5-point ordinal scale (1 = “Almost always”, 2 = “Often”, 3 = “Sometimes”, 4 = “Rarely”, 5 = “Almost never”). Perceived competencies, resources and motivation to provide smoking cessation support were rated on a 0–10 numeric scale (0 = “Not at all” to 10 = “Very much”).

### 3.6 Analyses

Analyses included logistic regression for smoking cessation at the 6-week follow-up and multiple regression for all other outcomes. Each analysis was conducted both unadjusted (with intervention group as the sole predictor) and adjusted (including age and, when possible, baseline levels of the respective outcome). Due to the small sample size in the study, we did not include additional covariates in the regression model. For logistic regression analyses, the chosen measure of association was odds ratio (OR) with 95% confidence interval (CI). For normal regression analyses, measure of association was the unstandardized difference between the level of control and intervention (i.e., *μ*_intervention_ - μ_control_) with 95% CI.

Cluster-robust standard errors accounted for patient clustering within hospital units. For logistic regression models, model fit was assessed using Pearson and Hosmer-Lemeshow goodness-of-fit tests, alongside manual inspection of predicted vs. observed counts across 10 quantiles. Multiple regression models were evaluated for homoscedasticity and normality of residuals via scatter plots and QQ-plots, revealing no violations of model assumptions.

Descriptive statistics of healthcare professionals' characteristics and outcomes in intervention vs. control groups from before to after the implementation period were computed and presented as bar charts. Because it was not possible to determine whether the same respondents participated before and after the implementation period, the assumption of independent observations could not be met. Therefore, no inferential statistical tests were conducted for these outcomes.

All analyses were conducted using Stata 18 (StataCorp LLC, College Station, TX, USA).

## 4 Results

After randomisation, five hospitals (Aalborg, Gentofte, Næstved, Odense and Vejle) formed the intervention group and the four remaining hospitals formed the control group (Aarhus, Bispebjerg, Roskilde and Sønderborg).

**Figure 1:**
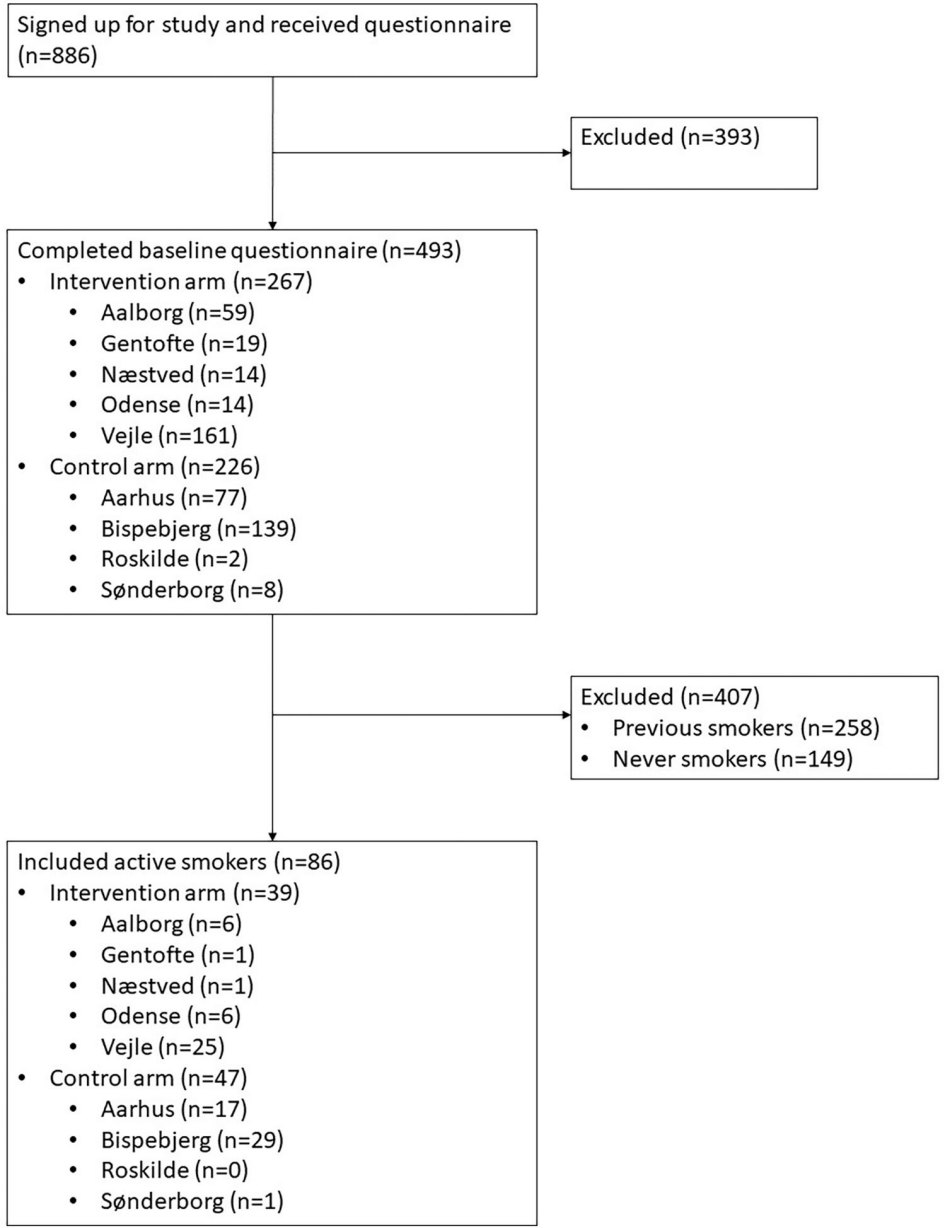
Patient flowchart.

In the period between March 2022 and June 2024 a total of 886 patients signed up for the study via the electronic patient letter. However, only 493 patients (56%) completed the questionnaire, and only 86 of these (17%) reported that they were active smokers at baseline, and hence, eligible for inclusion in the present study (Figure 1). This resulted in a total of 39 patients in the intervention group and 47 patients in the control group. An overview of patient characteristics at baseline can be found in Table 1. The number of patients who completed each outcome measure in the follow-up questionnaire varied, but responders and non-responders did not differ significantly at baseline with respect to any measured parameters (Table 2).

**Table 1:** Descriptive statistics by treatment condition (smoking cessation support implemented in usual care vs. usual care) for smokers undergoing lung cancer diagnostic workup at baseline.

Baseline characteristics	Intervention	Control	Total
*N* [*n* (%)]	39 (45.3%)	47 (54.7%)	86 (100.0%)
Age [Mean (SD)] [missing: 5 (5.8%)]	66.21 (11.07)	64.26 (8.79)	65.17 (9.91)
Gender [*n* (%)]			
Female	16 (41.0%)	24 (51.1%)	40 (46.5%)
Male	22 (56.4%)	22 (46.8%)	44 (51.2%)
Missing	1 (2.6%)	1 (2.1%)	2 (2.3%)
Number of cigarettes per day [Mean (SD)] [missing: 1 (1.2%)]	17.74 (13.69)	13.22 (12.58)	15.29 (13.22)
Age at smoking initiation [Mean (SD)] [missing: 6 (7.0%)]	14.97 (2.86)	15.11 (3.52)	15.05 (3.23)
Previous number of quit attempts [Mean (SD)] [missing: 16 (18.6%)]	4.90 (4.09)	3.46 (2.14)	4.10 (3.21)
Living with a partner who is smoking [*n* (%)]			
Yes	10 (25.6%)	10 (21.3%)	20 (23.3%)
No	15 (38.5%)	17 (36.2%)	32 (37.2%)
Have no partner	12 (30.8%)	18 (38.3%)	30 (34.9%)
Missing	2 (5.1%)	2 (4.3%)	4 (4.7%)
Nicotine dependence (Fagerstöm's test) [Mean (SD)] [missing: 12 (14.0%)]	3.69 (1.95)	3.05 (1.83)	3.36 (1.91)
Motivation to quit (VAS) [Mean (SD)] [missing: 1 (1.2%)]	58.41 (33.39)	65.87 (27.67)	62.45 (30.47)
Importance of quitting (VAS) [Mean (SD)] [missing: 1 (1.2%)]	65.05 (36.63)	77.67 (29.47)	71.88 (33.35)
Competencies to quit (VAS) [Mean (SD)] [missing: 1 (1.2%)]	58.72 (30.26)	66.17 (27.59)	62.75 (28.91)
Physical quality of life (SF-36) [Mean (SD)] [missing: 4 (4.7%)]	39.52 (11.26)	41.75 (10.85)	40.77 (11.02)
Mental quality of life (SF-36) [Mean (SD)] [missing: 4 (4.7%)]	43.12 (12.77)	44.40 (11.79)	43.84 (12.17)

SD, standard deviation; SF-36 (23); VAS, visual analogue scale.

**Table 2:** Differences at baseline for questionnaire responders vs. non-responders at 6 weeks follow-up after lung cancer diagnostic workup.

Baseline characteristics	Responders at follow-up			Non-responders at follow-up			*p*
	*N*	Mean/%	SD/IQR	*N*	Mean/%	SD/IQR	
Age	58	65.29	9.35	23	64.87	11.41	0.864
Smoking initiation (mean, SD)	57	14.81	3.46	23	15.65	2.55	0.292
Cigs per day (mean, SD)	46	17.28	16.25	39	12.95	7.93	0.133
Fagerström sum (mean, SD)	43	3.44	1.99	31	3.26	1.81	0.685
Motivation VAS (mean, SD)	44	61.25	28.87	41	63.73	32.42	0.710
Importance VAS (mean, SD)	44	75.14	30.02	41	68.39	36.64	0.355
Confidence VAS (mean, SD)	42	61.43	27.3	43	64.05	30.67	0.679
Physical quality of life (SF-36) (mean, SD)	56	40.14	11.07	26	42.13	10.99	0.451
Mental quality of life (SF-36) (mean, SD)	56	42.74	12.58	26	46.2	11.08	0.234
Female (*n*, %)	29	47.5%	-	11	47.8%	-	0.981
Attempts number (median, IQR)	51	3	2–5	19	3	2–5	0.179

IQR, interquartile range; SD, standard deviation; SF-36 (23); VAS, visual analogue scale.

### 4.1 Patient-reported outcomes

At follow-up, 60.7% of patients in the intervention group had initiated smoking cessation, compared with 65.7% in the control group. Unadjusted logistic regression analyses indicated lower, but statistically non-significant, odds for initiation of smoking cessation in the intervention group relative to the control group, OR  =  0.81 [0.41, 1.58], *p* = 0.53. The estimate was similar after adjusting for age, OR  =  0.79 [0.35, 1.79], *p* = 0.57 (Table 3). A total of 25% of patients in the intervention group reported to have stopped smoking entirely, compared with 28.6% in the control group, with no statistically significant difference between groups, OR  =  0.83 [0.50, 1.40], *p* = 0.49.

**Table 3:** Crude and adjusted effect of smoking cessation support implemented in usual care (intervention, *n* = 39) vs. usual care (control, *n* = 47) for smokers undergoing lung cancer diagnostic workup.

Outcomes	Unadjusted			Adjusted		
	OR/Diff intervention-control^a^	95% CI	*p*-value	OR/Diff intervention-control^a^	95% CI	*p*-value
Initiation of smoking cessation	0.81	[0.41, 1.58]	0.53	0.79	[0.35, 1.79]	0.57
Smoking cessation	0.83	[0.50, 1.40]	0.49	1.00	[0.52, 1.92]	1.00
Motivation to quit (VAS)	−12.19	[−41.35, 16.98]	0.33	−2.73	[−17.82, 12.35]	0.66
Importance of quitting (VAS)	−12.28	[−37.39, 12.83]	0.26	−1.10	[−11.19, 8.99]	0.79
Competencies to quit (VAS)	0.83	[−31.03, 32.69]	0.95	5.41	[−14.97, 25.78]	0.53
Physical quality of life (SF-36)	−1.03	[−9.55, 7.48]	0.77	1.09	[−6.63, 8.81]	0.73
Mental quality of life (SF-36)	1.19	[−5.74, 8.12]	0.68	1.72	[−3.61, 7.06]	0.44
Anxiety (COS)	−1.46	[−4.43, 1.51]	0.24	−1.30	[−4.53, 1.92]	0.32
Behaviour (COS)	−0.17	[−3.92, 3.58]	0.91	−0.03	[−3.57, 3.50]	0.98
Dejection (COS)	−0.23	[−3.78, 3.33]	0.87	−0.51	[−3.44, 2.42]	0.66
Negative impact on sleep (COS)	−1.69	[−2.62, −0.75]	0.01	−1.23	[−2.87, 0.41]	0.11
Existential (COS)	−0.87	[−2.40, 0.67]	0.21	−0.63	[−2.40, 1.13]	0.40
Relaxed/calm (COS)	−0.31	[−0.71, 0.09]	0.10	−0.25	[−0.74, 0.25]	0.26
Social relations (COS)	−0.13	[−0.74, 0.47]	0.59	−0.02	[−0.60, 0.56]	0.92

a Odds ratio reported for the primary outcome of Initiation of smoking cessation.

Mean difference (intervention-control) reported for all other outcomes. COS, consequences of screening—lung cancer questionnaire (24); OR, odds ratio; SF-36 (23); VAS, visual analogue scale.

**Figure 2:**
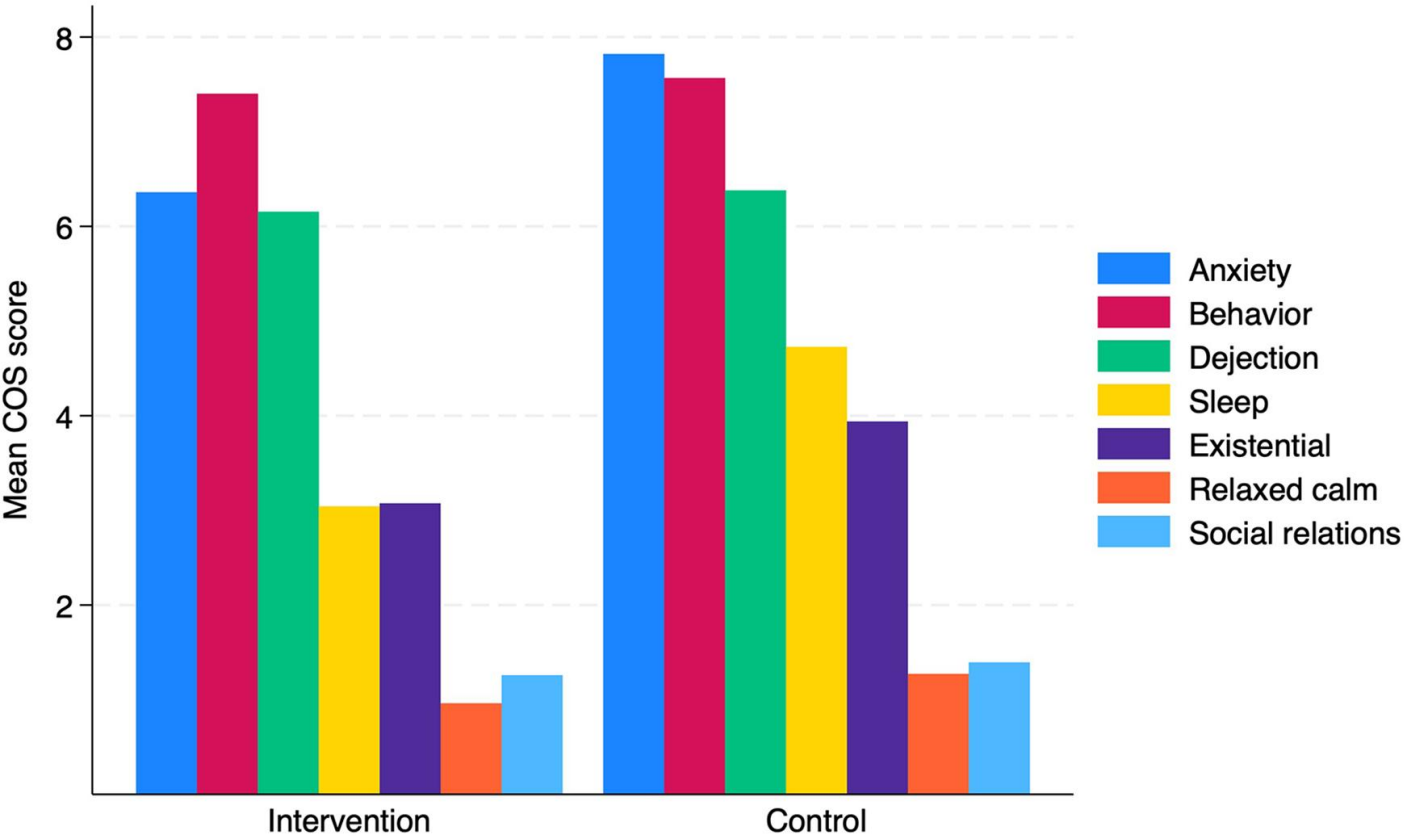
Patient-reported outcomes of smoking cessation support during lung cancer diagnostic workup on psychosocial consequences of diagnostic workup at 6 weeks follow-up in intervention (*n* = 39) and control (*n* = 47) conditions.

Unadjusted normal regression analyses showed that patients in the intervention group reported significantly lower impact of the diagnostic workup on sleep relative to the control group, *B* = −1.69 [−2,62, −0.75], *p* = 0.01. After adjusting for age, no statistically significant differences were seen (Table 3). No significant differences were found for the any of the remaining subdomains of the COS Questionnaire (Figure 2).

**Figure 3:**
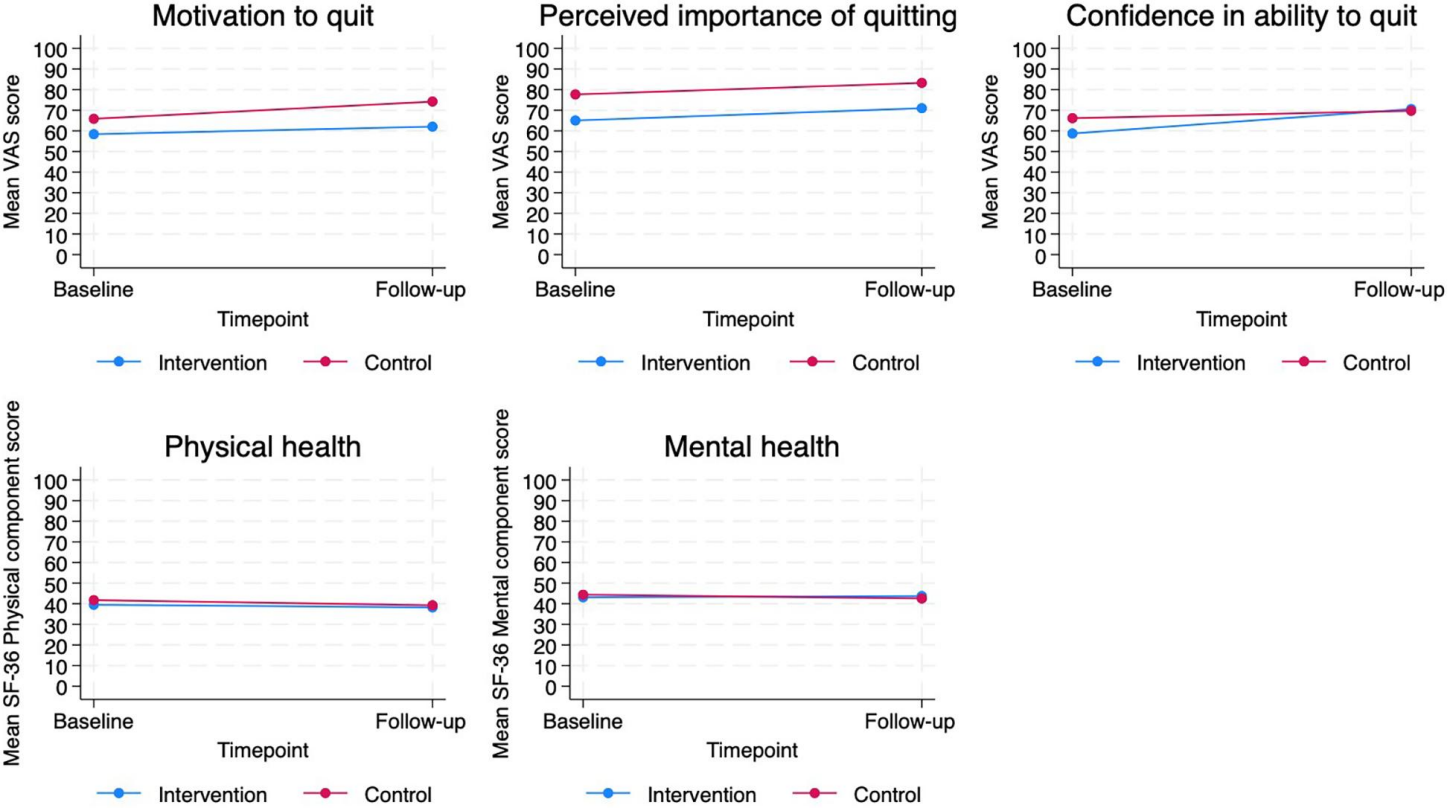
Patient-reported outcomes of smoking cessation support on the outcomes of motivation, importance and perceived competencies to quit, as well as quality of life, from baseline (beginning of lung cancer diagnostic workup) to 6 weeks follow-up in intervention (*n* = 39) and control (*n* = 47) conditions.

No statistically significant differences were found for the remaining outcomes (Figure 3).

### 4.2 Healthcare professionals-reported outcomes

Across all included hospitals, a total of 140 healthcare professionals completed the survey prior to implementation of the intervention, and 54 healthcare professionals completed the survey after the implementation (Table 4).

**Table 4:** Descriptive statistics of healthcare professionals at the participating hospitals before and after the implementation of smoking cessation support for smokers undergoing lung cancer diagnostic workup.

Baseline characteristics	Before implementation	After implementation	Total
*N*	140	54	194
Age [Mean (SD)]	42.99 (12.17)	46.69 (10.86)	44.02 (11.91)
Gender [*n* (%)]			
Female	109 (77.9%)	47 (87.0%)	156 (80.4%)
Male	31 (22.1%)	7 (13.0%)	38 (19.6%)
Work type [*n* (%)]			
Nurse	78 (55.7%)	41 (75.9%)	119 (61.3%)
Medical doctor	62 (44.3%)	13 (24.1%)	75 (38.7%)
Years employed in current organisation [Mean (SD)]	6.88 (8.17)	8.80 (7.92)	7.41 (8.13)
Work place [*n* (%)]			
Aalborg (intervention site)	37 (26.4%)	3 (5.6%)	40 (20.6%)
Aarhus (control site)	23 (16.4%)	9 (16.7%)	32 (16.5%)
Bispebjerg (control site)	9 (6.4%)	7 (13.0%)	16 (8.2%)
Gentofte (intervention site)	13 (9.3%)	10 (18.5%)	23 (11.9%)
Næstved (intervention site)	6 (4.3%)	9 (16.7%)	15 (7.7%)
Odense (intervention site)	27 (19.3%)	7 (13.0%)	34 (17.5%)
Roskilde (control site)	13 (9.3%)	0 (0.0%)	21 (10.8%)
Sønderborg (control site)	0 (0.0%)	0 (0.0%)	0 (0.0%)
Vejle (intervention site)	12 (8.6%)	9 (16.7%)	21 (10.8%)

SD, standard deviation.

**Figure 4:**
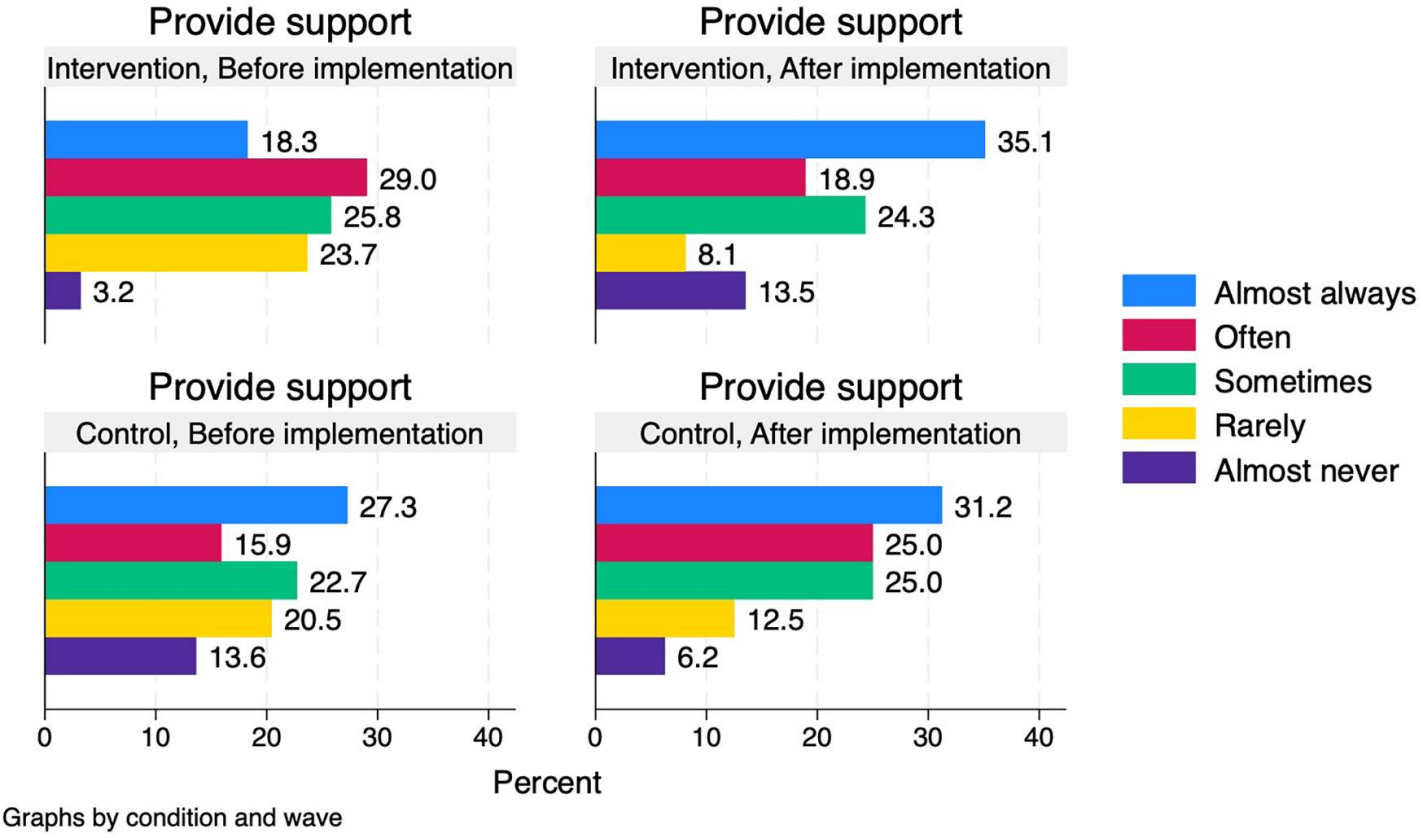
Healthcare professionals’ self-reported provision of smoking cessation support before and after implementation period in intervention and control conditions.

The proportion who reported that they “almost always” provided smoking cessation support was larger after (35.1%) than before (18.3%) the implementation of the intervention among healthcare professionals in the intervention group, but the proportion who reported to “almost never” provide support was also larger after (13.5%) than before (3.2%). In the control group, the proportions more generally tended to shift towards providing support more often (Figure 4).

**Figure 5:**
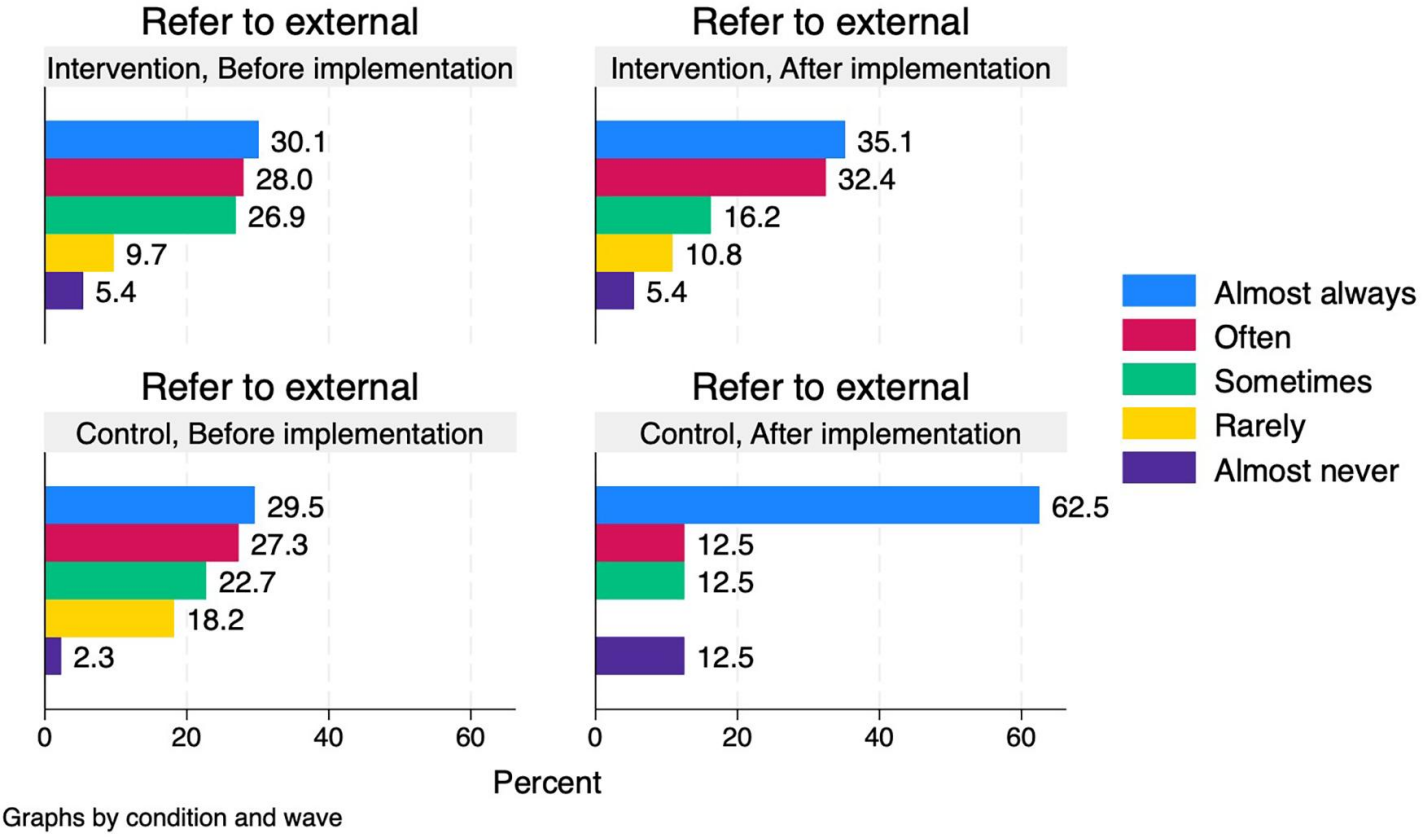
Healthcare professionals’ self-reported referral to external smoking cessation programmes before and after implementation period in intervention and control conditions.

Overall, the proportions of healthcare professionals who reported referring patients to external smoking cessation support were largely similar before and after the implementation period. However, in the control group, a substantially larger proportion of healthcare professionals reported that they “almost always” referred patients to external support at follow-up (62.5%) compared to the baseline measurement (29.5%) (Figure 5).

**Figure 6:**
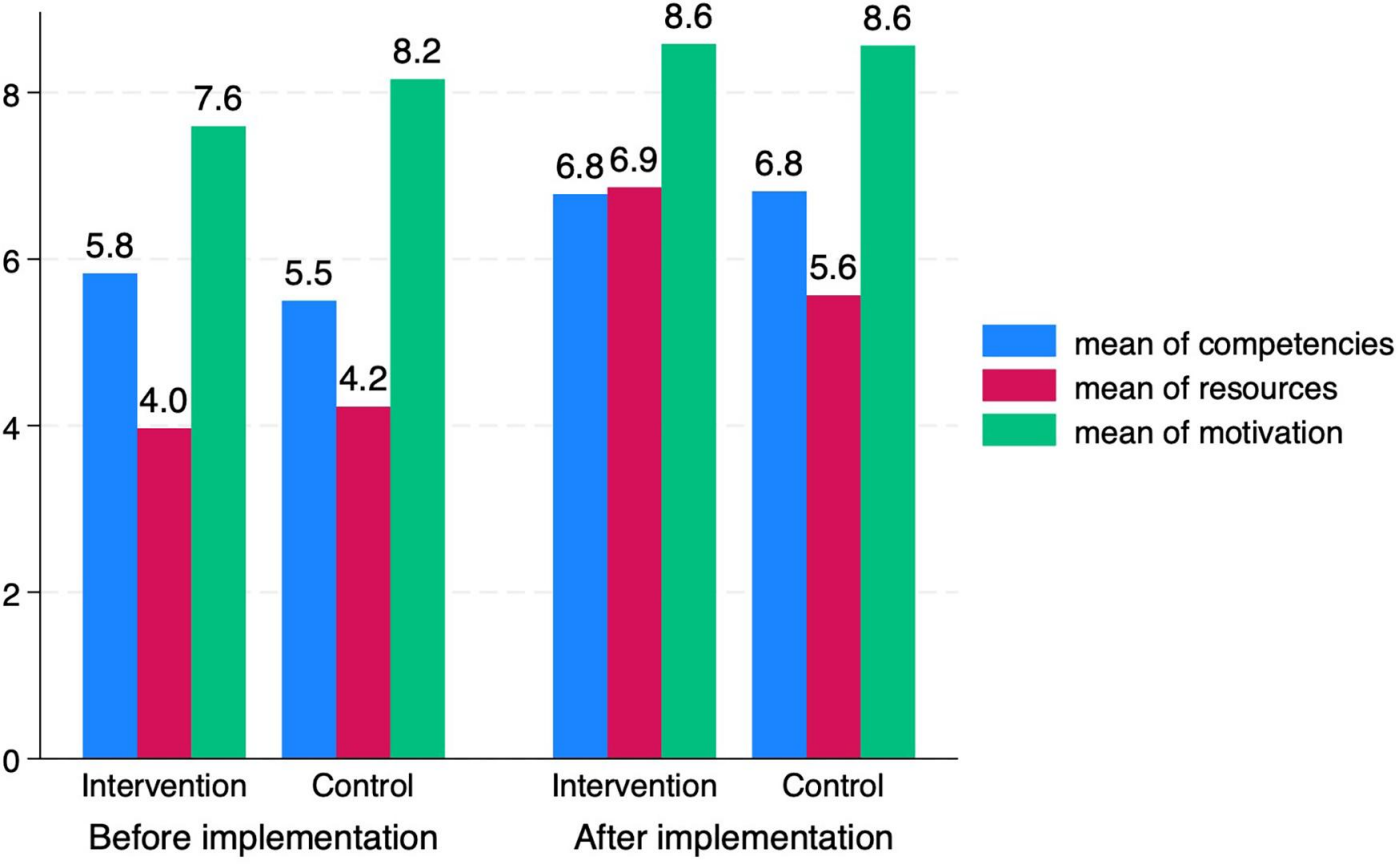
Healthcare professionals’ self-reported levels of competencies, resources and motivation to provide smoking cessation support before and after implementation period in intervention and control conditions.

We observed that average levels of self-reported competencies, resources and motivation to provide smoking cessation support were higher in both the intervention and control group after the implementation period, compared with before implementation (Figure 6). While the average levels of competencies and motivation were comparable in the intervention and control groups after the implementation, the level of resources were higher in the intervention group than in the control group.

## 5 Discussion

This pragmatic, multi-centre trial provides a practical framework for integrating structured smoking cessation support into the lung cancer diagnostic pathway. By embedding training, resources and referral procedures into existing workflows, the study demonstrates how cessation support can be operationalised in routine care. However, in evaluating this approach, we found no statistically significant effect of the intervention on the proportion of patients who initiated smoking cessation (intervention = 60.7%, control = 65.7%, adjusted OR = 0.79, *p* = 0.57) or who stopped smoking entirely (intervention = 25.0%, control = 28.6%, OR = 0.83, *p* = 0.49. Moreover, the findings are likely subject to substantial response bias, as only 17% were active smokers—and thus, eligible for inclusion—compared with the expected 40%. The effects on healthcare professionals’ practices were also inconclusive: At the intervention sites, a greater proportion of healthcare professionals reported always providing smoking cessation support after implementation and indicated improved availability of resources to facilitate this activity. But at the same time, a higher proportion also reported never providing such support after the implementation. In contrast, at the control sites, a substantially larger proportion of healthcare professionals reported always referring patients to external smoking cessation support at follow-up, while these proportions remained relatively unchanged at the intervention sites.

The similar rates of cessation initiation observed in the intervention (60.7%) and control groups (65.7%) are consistent with previous research in respiratory and serious health conditions indicating that the period surrounding diagnosis in itself represents a “teachable moment” for behaviour change. For instance, a Danish prospective population-based study found higher cessation rates following incident heart disease or asthma (26). A qualitative study among patients with chronic obstructive pulmonary disease (COPD) further suggests that guilt and shame linked to smoking may intensify when disease is suspected or diagnosed, potentially motivating behaviour change (27). While such feelings can be distressing, they may also bear the potential to catalyse a re-evaluation of health beliefs and increase readiness to quit, which may result in dissolving of the difficult feelings (28). In a systematic review examining smoking cessation at or around the time of lung cancer diagnosis (7), reported quit rates ranged from 17% to 95%, probably due to various definitions of cessation and the time frames considered “around diagnosis”. In the present study, 25% of patients in the intervention group and 28.6% in the control group reported having quit smoking entirely. Thus, both the proportion initiating cessation and the proportion achieving abstinence were at the lower end of the range reported in previous studies. This finding suggests that brief, targeted training combined with supportive written materials may not be sufficient to produce sustained behavioural change among healthcare professionals or meaningful improvements in patient cessation outcomes within complex clinical settings.

In the present study, the results did not reach statistical significance and there was indication of selection bias, underscoring the challenges of achieving non-biased samples of a sufficient size in heterogeneous, real-world clinical settings. Recruitment patterns are likely to have influenced results: the proportion of current smokers in the present study (17%) was markedly lower than in existing studies (40%–50%) (11, 12, 14), possibly due to the self-enrolment process via an electronic hospital letter without active recruitment by staff. This interpretation is supported by a qualitative study in which patients reported that their ability to use electronic devices declined during diagnostic workup, regardless of their prior technical skills, which they attributed to the emotional strain and stress of the situation (29). Conversely, another study found that computer-based reported of smoking status was accurate and acceptable even among disadvantaged populations (30). In addition, social desirability bias—the tendency for participants to provide responses they perceive as socially acceptable—and societal stigma surrounding smoking may have contributed to underreporting of active smoking. However, several large, longitudinal studies have not found associations between social desirability and self-reported health risk behaviours in web-based research (31). Although extending the recruitment period could have increased the sample size required by our *a priori* power analysis, we chose not to do so, as this would most likely not have resolved the response bias reflected in the low proportion of smokers among participants. In addition, prolonging recruitment would have increased the risk of enrolling patients exposed to an intervention that may have changed over time due to the nature of real-world implementation. Participants in the intervention group reported smoking a greater number of cigarettes per day and had a higher number of previous quit attempts compared to those in the control group. This suggests that the intervention group may have represented a more nicotine-dependent sample, which could have contributed to an underestimation of the intervention's effects. Alternatively, the higher number of prior quit attempts among participants in the intervention group may also be interpreted as an indicator of greater motivation to quit. Due to the limited sample size, we were unable to adjust for these potential covariates in our model. In unadjusted analyses, patients in the intervention group reported a lower negative impact of diagnostic workup on sleep. This could reflect the psychological benefits of taking proactive steps—such as quitting smoking—to improve health, potentially reducing worry and promoting better sleep, which resonates with findings from a lung cancer screening study where cessation was linked to “a peace of mind about the disease” (32). Biological pathways linking smoking to sleep disturbance, including metabolic, neurological and inflammatory mechanisms, may also contribute (33). However, this association was attenuated after adjusting for age and should therefore be interpreted with caution.

A common concern among healthcare professionals is that discussing smoking may distress patients during an already stressful period (17). In a study of smoking and pain coping, smoking was used as a cognitive distraction to reduce distress, despite paradoxically worsening pain intensity (34). A longitudinal lung cancer study applying the self-regulation model of illness found that newly diagnosed smokers actively construct personal “illness representations” to make sense of their condition, e.g., what it is, what it means, its causes, consequences, duration and controllability (35). These representations are shaped by symptoms, emotions, social influences and interactions with healthcare providers, and are not always medically accurate, but can strongly influence health behaviour, including smoking. Importantly, the present study found no evidence that providing cessation support increased the perceived burden of the diagnostic process or worsened quality of life.

In addition to the lack of effect on smoking cessation *behaviour*, the intervention did not significantly change patients' *motivation* to quit or perceived importance of cessation—factors typically being considered precursors to behaviour change within the Stages of Change model (36, 37). This may be due to the characteristics of the recruited sample, which had relatively high baseline levels of motivation, importance and self-perceived competence to quit, as well as an average of four prior quit attempts. These characteristics suggest that some might have already prepared to quit and may have required only the contextual cue of a lung cancer workup (12), whereas others might have been motivated, but were not capable of acting on this intention due to high addition levels and stress. Moreover, the proportion of patients who reported complete smoking cessation was considerably lower than the proportion who reported having initiated cessation efforts. This further highlights the processual nature of smoking cessation, in which the preparation stage generally precedes action and maintenance (22, 36). A study design incorporating longer follow-up and tracking of engagement with community-based cessation services could have provided better insight into individual cessation trajectories. It should be noted, that those less motivated or less aware of cessation benefits—a group potentially more responsive to targeted support—were likely underrepresented in the present study due to the aforementioned recruitment-related selection bias. Their exclusion may have limited the intervention's ability to demonstrate an effect on motivation or cessation outcomes.

Descriptively, the effects of the intervention on healthcare professionals' practices were mixed. Following implementation, a larger proportion of healthcare professionals at the intervention sites reported providing smoking cessation support more consistently and noted improved access to supporting resources, while others reported never providing such support. This suggests that relatively brief, targeted training may influence how healthcare professionals perceive their opportunities to deliver smoking cessation support in clinical practice, although this did not translate into consistent behaviour change or measurable effects on patient smoking cessation behaviours.

Interestingly, referral to external smoking cessation support increased substantially in the control sites, whereas little change was observed at the intervention sites. This may reflect a broader societal trend toward heightened awareness of smoking cessation support in healthcare (38, 39), where healthcare professionals in the control group—who did not receive training themselves—may have responded by referring more patients to external services. However, it remains unclear whether these referrals resulted in greater uptake of external support or whether patients ultimately benefitted from such services.

According to the COM-B model, increases in perceived competence, opportunity and motivation are drivers of behaviour change (40). This was only partly reflected in our findings, which showed a higher level of healthcare professionals' self-reported competence, resources and motivation to provide support after the implementation period, but not a clear translation into actually offering cessation advice and referring patients to external programmes. However, these results should be interpreted with caution, as no statistical testing was performed due to changes in staff composition before and after implementation, and because hospitals were unequally represented across measurement points, which may have influenced implementation fidelity and provider outcomes. Moreover, a markedly lower number of healthcare professionals completed the survey after the implementation period (*n* = 54), compared with before (*n* = 140). Due to the anonymity in data collection the proportion of individual participants who responded to the survey both before and after the implementation period is unknown, and the apparent positive changes could be due to selection bias where those who were more motivated to implement smoking cessation support were also more inclined to complete the follow-up questionnaire. Ongoing evaluation and non-anonymised data collection will be important to determine whether changes after such implementations can be sustained over time, particularly after staff turnover and with shifting institutional priorities.

### 5.1 Strengths and limitations

A strength of this study is its pragmatic, multi-centre design, which enhances the external validity and applicability of the findings across different hospital settings. By embedding the intervention within the existing lung cancer diagnostic pathway, the study reflects real-world clinical conditions and provides a concrete framework for implementing smoking cessation support in routine practice. The use of cluster randomisation at the hospital level minimised contamination between intervention and control groups and preserved the integrity of the training components. The focus on both patient and healthcare professional outcomes allowed for a more comprehensive understanding of the potential impact of the intervention. Additionally, the intervention was grounded in evidence-based clinical guidelines and supported by structured training and written resources, which increasing its relevance and scalability in policy and practice.

Several limitations should be considered when interpreting the results. First, the proportion of active smokers among patients who signed up for the study was substantially lower than anticipated, suggesting response bias related to the recruitment method (electronic self sign-up). This approach was chosen to ensure that evaluation procedures did not take away healthcare professionals’ time and focus from the implementation process. With the purpose of increasing number of participants in future studies, the core research team could provide telephone reminders with the option to respond via structured telephone interviews if they have difficulty with online completion of questionnaires.

Second, although the projected number of respondents was enrolled, the lower-than-expected prevalence of current smokers resulted in an underpowered final sample, which may have contributed to non-significant findings. We did not extend the study period, as prolonged recruitment could have increased heterogeneity in intervention exposure due to the evolving nature of implementation. Additionally, the inclusion criterion requiring participants to be active smokers may have excluded individuals who had very recently initiated a quit attempt—who could therefore also have benefited from cessation support given the high risk of relapse in the days and months following a quit attempt.

Third, smoking cessation outcomes were based on self-report without biochemical validation (e.g., cotinine testing), which may have introduced reporting bias. However, as our primary outcome was initiation of smoking cessation rather than abstinence, this limitation is likely to have had less influence. Moreover, a systematic review and meta-analysis have shown that self-reported smoking status is generally reliable, particularly in older populations (41). The choice of initiation of cessation as the primary outcome aligns with implementation-focused and hospital-based cessation studies, which commonly evaluate proximal indicators such as referral, enrolment and engagement with cessation support services (42–44). Such proximal effects are what the intervention of the present study was designed to produce. Measuring initiation therefore captures the immediate, clinically meaningful behavioural response to hospital-based encounters, while also recognising that sustained abstinence typically requires longer follow-up and multiple quit attempts.

Fourth, although cluster randomisation reduced the risk of contamination, differences between hospitals in patient characteristics, local resources and workflow organisation may have influenced the results. The pragmatic design further limited assessment of intervention fidelity, as provider engagement and patient exposure were not formally monitored. To minimise the impact of this, standardised training and ongoing supervision were provided to healthcare professionals at intervention sites. However, we did not monitor the proportion of healthcare professionals at the intervention sites who actually participated in the training, and, due to the anonymity of survey responses, we were unable to asses the characteristics of non-responding staff. As a results, it is unclear whether the training reached a representative sample of staff, which may limit the generalisability of the findings and the interpretation of the intervention's effectiveness at the organisational level.

Finally, the primary endpoint was assessed 6 weeks after baseline, capturing the immediate post-diagnostic period, but not long-term cessation maintenance. Nevertheless, a recent meta-analysis of hospital-initiated cessation support suggests that intervention effects are generally sustained over time (45). The outcome of the diagnostic assessment—whether a lung cancer diagnosis was confirmed or not—may have influenced the results. For example, a qualitative study reported that patients that did not receive a lung cancer diagnosis perceived their smoking as less harmful (46). As we did not collect data on participants' diagnostic outcomes, we were unable to explore this further.

### 5.2 Future directions

Future research should investigate the implementation effect of hospital-based smoking cessation support with particular emphasis on achieving unbiased inclusion of current smokers. Additionally, strategies to improve both uptake and sustained delivery of cessation support throughout the lung cancer care pathway should be explored. Given that smoking cessation is often a prolonged process requiring multiple attempts, continuity of support beyond diagnosis is essential. Implementation studies guided by frameworks such as RE-AIM (47) could examine barriers and facilitators at individual, team, departmental and organisational levels. Potential enhancements include digital tools to facilitate patient–provider communication and systematic involvement of caregivers in cessation strategies. Identifying and engaging less-motivated smokers—potentially the group most in need of targeted intervention—should be a priority.

## 6 Conclusion

This study was inconclusive, as it did not demonstrate statistically significant effects of providing smoking cessation support during lung cancer diagnostic workup on patients' initiation of smoking cessation. These findings may have been influenced by selection bias and variations in implementation fidelity. Similarly, observed changes in healthcare professionals' practices were mixed: although the intervention may have increased awareness and enhanced perceptions of capacity to provide smoking cessation support, this did not translate into consistent behavioural change. Overall, the results suggest that brief, targeted training and supportive written materials alone may be insufficient to achieve sustained changes in practice within a complex clinical setting.

## Data Availability

The raw data supporting the conclusions of this article will be made available by the authors, without undue reservation.
