# Supplementary figures and images for: Integrating smoking cessation support during lung cancer diagnostic workup: a pragmatic, multicenter, cluster-randomised controlled trial
Source: Front Health Serv. 2025 Dec 9;5:1696454. doi: 10.3389/frhs.2025.1696454 (PMC12722788; doi:10.3389/frhs.2025.1696454)

**Figure 1. Smoking cessation support during lung cancer diagnostic workup (overview)**

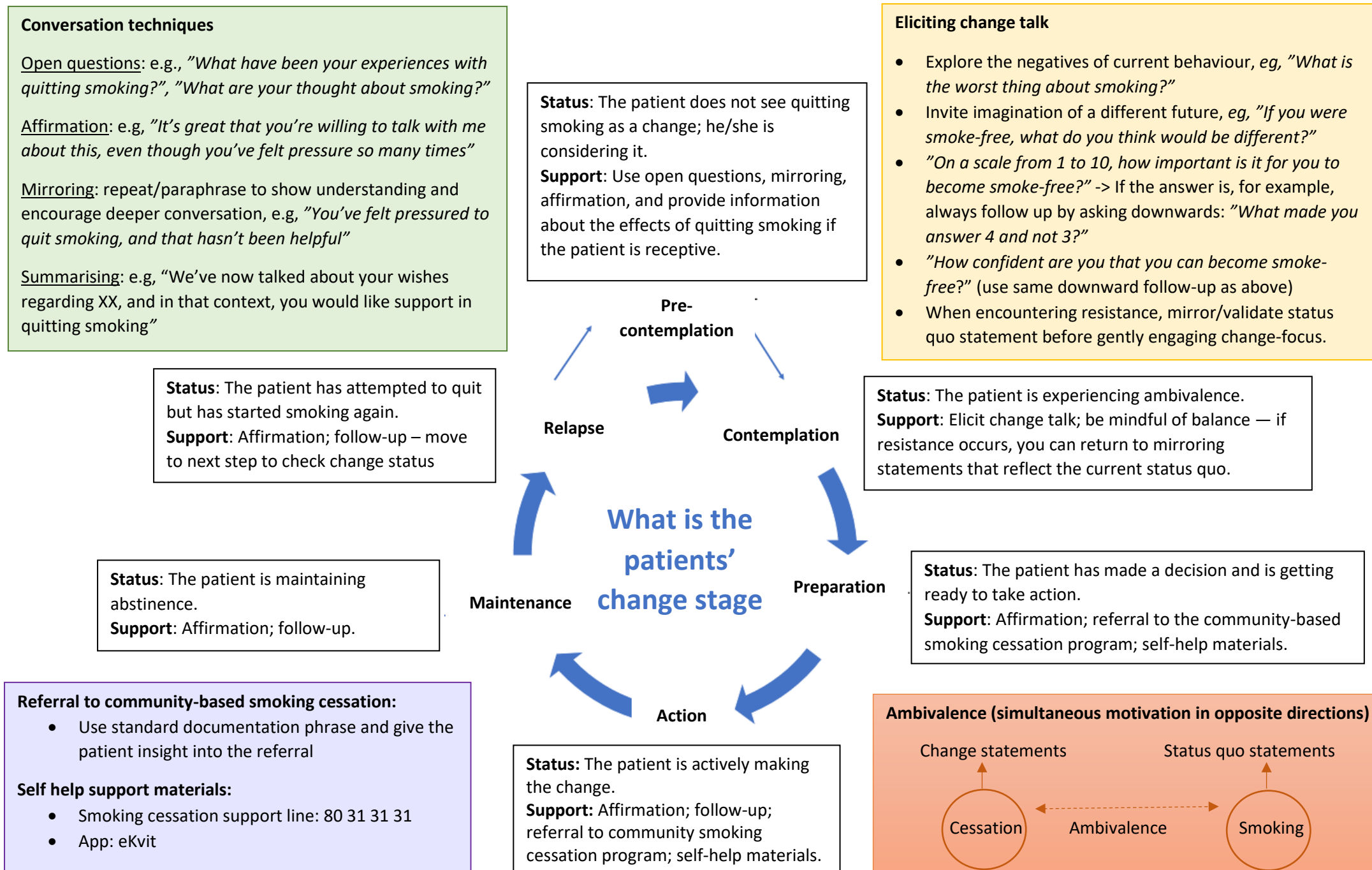

Supplement: Supplementary file 1 [file Datasheet1.pdf]
