# Supplementary material for: Integrating smoking cessation support during lung cancer diagnostic workup: a pragmatic, multicenter, cluster-randomised controlled trial
Source: Front Health Serv. 2025 Dec 9;5:1696454. doi: 10.3389/frhs.2025.1696454 (PMC12722788; doi:10.3389/frhs.2025.1696454)
Supplement: Supplementary file 2 [file Datasheet2.pdf]

```
. Figure 4 - PROVIDE SUPPORT
. tab condition cessation_initiation if wave == 1,mis row

+----------------+
| Key            |
|----------------|
|   frequency    |
| row percentage |
+----------------+

             |
             |
   condition | Almost al      Often  Sometimes     Rarely  Almost ne          .
|     Total
-------------+------------------------------------------------------------------
+----------
Intervention |        17         27         24         22          3          2
|        95
             |     17.89      28.42      25.26      23.16       3.16       2.11
|    100.00
-------------+------------------------------------------------------------------
+----------
     Control |        12          7         10          9          6          1
|        45
             |     26.67      15.56      22.22      20.00      13.33       2.22
|    100.00
-------------+------------------------------------------------------------------
+----------
       Total |        29         34         34         31          9          3
|       140
             |     20.71      24.29      24.29      22.14       6.43       2.14
|    100.00

. tab condition cessation_initiation if wave == 2,mis row

+----------------+
| Key            |
|----------------|
|   frequency    |
| row percentage |
+----------------+

             |
             |
   condition | Almost al      Often  Sometimes     Rarely  Almost ne          .
|     Total
-------------+------------------------------------------------------------------
+----------
Intervention |        13          7          9          3          5          1
|        38
             |     34.21      18.42      23.68       7.89      13.16       2.63
|    100.00
```

```
-------------+------------------------------------------------------------------
+----------
     Control |         5          4          4          2          1          0
|        16
             |     31.25      25.00      25.00      12.50       6.25       0.00
|    100.00
-------------+------------------------------------------------------------------
+----------
       Total |        18         11         13          5          6          1
|        54
             |     33.33      20.37      24.07       9.26      11.11       1.85
|    100.00

. Figure 5 - REFERRAL
. tab condition cessation_referral if wave == 1,mis row

+----------------+
| Key            |
|----------------|
|   frequency    |
| row percentage |
+----------------+

             |
             |
   condition | Almost al      Often  Sometimes     Rarely  Almost ne          .
|     Total
-------------+------------------------------------------------------------------
+----------
Intervention |        28         26         25          9          5          2
|        95
             |     29.47      27.37      26.32       9.47       5.26       2.11
|    100.00
-------------+------------------------------------------------------------------
+----------
     Control |        13         12         10          8          1          1
|        45
             |     28.89      26.67      22.22      17.78       2.22       2.22
|    100.00
-------------+------------------------------------------------------------------
+----------
       Total |        41         38         35         17          6          3
|       140
             |     29.29      27.14      25.00      12.14       4.29       2.14
|    100.00

. tab condition cessation_referral if wave == 2,mis row

+----------------+
| Key            |
|----------------|
|   frequency    |
| row percentage |
+----------------+
```

```
             |
             |
   condition | Almost al      Often  Sometimes     Rarely  Almost ne          .
|     Total
-------------+------------------------------------------------------------------
+----------
Intervention |        13         12          6          4          2          1
|        38
             |     34.21      31.58      15.79      10.53       5.26       2.63
|    100.00
-------------+------------------------------------------------------------------
+----------
     Control |        10          2          2          0          2          0
|        16
             |     62.50      12.50      12.50       0.00      12.50       0.00
|    100.00
-------------+------------------------------------------------------------------
+----------
       Total |        23         14          8          4          4          1
|        54
             |     42.59      25.93      14.81       7.41       7.41       1.85
|    100.00

.
. log close
      name:  <unnamed>
       log:  /Users/au258406/Desktop/Projekter personer/Ingeborg START
(rygestop-projekt)/Data og analyser/counts for figure 4 and 5.log
  log type:  text
 closed on:  12 Nov 2025, 10:57:59
--------------------------------------------------------------------------------
--------------------------------------------------------------------------------
--------------
```
