# Supplementary material for: Integrating smoking cessation support during lung cancer diagnostic workup: a pragmatic, multicenter, cluster-randomised controlled trial
Source: Front Health Serv. 2025 Dec 9;5:1696454. doi: 10.3389/frhs.2025.1696454 (PMC12722788; doi:10.3389/frhs.2025.1696454)
Supplement: Supplementary file 3 [file Table1.docx]

| **Table 1 – Supplementary material** |
| --- |
| **Patient and Family Information** **Smoking Cessation During Lung Cancer Diagnostic Workup**  Read this brief information leaflet whether or not you plan to quit smoking. It will help you understand the risks of continued smoking as well as the benefits of quitting.  It is important for your lungs and overall health to stop smoking, regardless of the results of your examinations with us. Quitting can also reduce the risk of complications during any tests or procedures you may undergo while under our care.  **Our staff will discuss smoking with you.**  **Immediate Benefits of Quitting Smoking**  Within hours and days after your last cigarette, your body begins a gradual detoxification, and you may notice improvements relatively quickly:   - **20 minutes:** Blood pressure and heart rate normalize; circulation improves. - **8 hours:** Carbon monoxide levels in your blood drop; your oxygen levels improve. - **24 hours:** Risk of heart attacks and respiratory issues decreases. - **48 hours:** Carbon monoxide from tobacco is eliminated from your body. - **2–3 days:** Less or no phlegm in your throat; easier breathing. - **5–7 days:** More energy; improved sense of taste and smell; fresher breath; cleaner teeth. - **2–3 weeks:** Nicotine withdrawal symptoms subside, and you can go hours without thinking about smoking.   **Long-Term Benefits of Quitting Smoking**  The long-term benefits of quitting are substantial, even though quitting may seem challenging and cause short-term discomfort.  **Benefits include:**   - Reduced risk of lung disease - Reduced risk of worsening existing lung conditions - Reduced risk of cardiovascular events and cancer complications - Improved quality of life - Improved mood   **Quitting Smoking Reduces the Risk of Complications**  Stopping smoking helps reduce the risk of complications during examinations and anesthesia related to your lung assessment:   - Smoking damages the lungs and increases the risk of breathing problems and infections during procedures (e.g., tissue biopsies). - Smoking increases the risk of wound infections.   **If Lung Cancer is Diagnosed**  If your evaluation with us shows that you have lung cancer, quitting smoking becomes a very important part of your treatment.  **Benefits of quitting if you have lung cancer:**   - Longer life expectancy - Better effectiveness and fewer side effects from chemotherapy and radiation - Reduced risk of disease spread - Improved quality of life - Reduced risk of complications from surgery - Fewer wound complications - Fewer lung and cardiovascular complications after surgery - Shorter hospital stays - Reduced risk of death related to surgery - Reduced risk of cancer recurrence   **Support for Quitting Smoking**  Whether you have tried to quit many times before or are attempting it for the first time, it is natural to need help and support to stay smoke-free.  Our staff will discuss smoking with you, and additional help is available from:   - Your general practitioner (GP) - Local community-based programs - Your pharmacy - **Stoplinien** (Danish Quitline) – tel. 80 31 31 31 - The **e-Kvit** app   Our staff can refer you to a support program if you wish.  **Tip:** Write down your questions here – it helps you remember them! |
