# Supplementary material for: Integrating smoking cessation support during lung cancer diagnostic workup: a pragmatic, multicenter, cluster-randomised controlled trial
Source: Front Health Serv. 2025 Dec 9;5:1696454. doi: 10.3389/frhs.2025.1696454 (PMC12722788; doi:10.3389/frhs.2025.1696454)
Supplement: Supplementary file 4 [file Table2.docx]

| **Table 2 – Supplementary material** |
| --- |
| **Referral note template for electronic patient records:**  “[Name] is currently undergoing evaluation due to a justified suspicion of lung cancer. A smoking cessation program should therefore be initiated as soon as possible due to the specific current health risks associated with continued smoking. Please context [Name] as soon as possible by phone at [phone number].”  **Ensure the patient is informed about the referral.** |
